# Therapeutics

**Published:** 1877-02

**Authors:** 


### IV. Therapeutics.

1. *Glycerole of Nitrate of Bismuth.* Squire. (*Pharm. Journal.*)

Dr. Squire finds that nitrate of bismuth is freely soluble in glycerine without the addition of water, and suggests its use as a bland and mild astringent in diseases of the throat, larynx, vagina, uterus and urethra.

J. S. K.

2. *Bromide of Potassium in Hæmorrhage.* (*New Remedies.*)

It has been asserted that this agent induces contraction of the blood-vessels, and Dr. Genenal (*L'Union Médicale*) has used a local application in the treatment of epistaxis, uterine hæmorrhage and coryza. It should also be given internally. In coryza two injections of a saturated solution into the nose give relief in an hour, and a permanent cure is effected in about six hours. J. S. K.

3. *Antidotes of Arsenic.* Rouyer. (*New Remedies. Canadian Journal.*)

R. as the result of many experiments publishes the following:

1. Hydrated sesquioxide of iron, recently prepared, (gelatinous and brown) is an antidote for arsenious acid, but not for arseniate of potash nor for the arseniate of soda.

2. At a longer interval than an hour it is useless to attempt recovery from poisoning by arsenic.

3. For arsenite of potash and arsenite of soda the author proposes perchloride of iron in conjunction with magnesia.

4. The mode of administration is the officinal solution of perchloride of iron, and half an hour afterward magnesia in the proportion of a drachm to 3¼ fl. ozs. of perchloride.

5. This of perchloride iron and magnesia is also an antidote for arsenious acid. Therefore, it is preferable to employ it always in cases of poisoning by arsenic or its compounds.

6. An hour after the administration of an antidote it will always be well to employ a purgative, in order to expel the ferrated arsenic which is formed, and as this arsenite is soluble in acids to avoid acid drinks and lemonades. J. S. K.

4. *Emulsion of Cod Liver Oil.* (*Druggists' Circular.*)

| | | |
|---|---|---|
| Cod liver oil | 4 | ounces. |
| Glyconine | 9 | drachms. |
| Aromat. spts. ammon | 1 | " |
| Phosphoric acid dilut | 4 | " |
| Essense bitter almonds | 2 | " |
| Sherry wine or brandy | 16 | " |

*Glyconine* is made by adding five parts, by weight, of concentrated glycerine to four parts of yolks of eggs well beaten. J. S. K.

5. *Strong Solution of Salicylic Acid.* (*New Remedies.*)

| | |
|---|---|
| Salicylic acid | ʒ j |
| Potassium, sodium, or ammonium acetate | ʒ j |
| Glycerine | ʒ j |
| Water q. s. ad | ℥ j |

Mix and dissolve by shaking. This solution contains one grain of salicylic acid in eight minims, and is largely used in Bellevue and other eastern hospitals. J. S. K.

6. *Bromide of Lithium.* (*Southern Med. Record.*)

Dr. Rouband writes in the *Revue de Thérapeutique* as follows of this drug: "1. Bromide of Lithium is a drug which has a twofold action. 2. It possesses in a high degree the lithontriptic qualities which are universally recognized in the salts of lithia. 3. It affects reflex sensibility in a more energetic manner than the other bromides, without the unpleasant effects upon the heart which the bromide of potassium has. 4. Consequently it takes its place in the first rank of antilithic and sedative drugs, and its action is especially valuable in cases of the uric acid diathesis which are accompanied by painful phenomena." J. S. K.

7. *Liquid Bismuth or Elixir Bismuth.*

The following is the formula adopted by the American Pharmaceutical Association:

| | |
|---|---|
| Ammonio citrate of bismuth | 256 grs. |
| Warm distilled water | 1 fl. ounces. |
| Water of ammonia sufficient to neutralize simple elixir | 15 fl. " |

Mix and filter. J. S. K.

8. *Nitrite of Amyl.*

Nitrite of amyl has been tried, says the *Dublin Journal*, both in corea and intermittent fever. In corea inhalations of from 3 to 6 drops were order three times a day, arresting the convulsions in three cases at the end of two weeks. In intermittent fever it has aborted the chill and shortened the latter stages, but does not prevent a recurrence. Thirty drops have been administered as a dose with advantage in this fever. J. S. K.

9. *A New Anti-Pruritic Remedy.* BULKLEY. (*Trans. Amer. Med. Asso.*)

Dr. B. offers to the profession the following formula:

| | | |
|---|---|---|
| ℞ Pulv. Gummi Camph. | *aa* | ʒ j |
| Chloral Hydrate. | | |
| Ung. Aquae Rosæ | | ℥ j |

M.

Rub the chloral and camphor carefully together till fluid results, then add slowly the ointment, mixing well.

This, when applied to the healthy skin produces no effect; but possesses great power in arresting itching without over stimulating the parts. It does not answer when the skin is at all broken; it is then necessary to employ other less irritating agents; but the burning sensation caused on its first application lasts but a few moments, while the relief occasioned will last for hours, or even a whole day. J. S. K.

10. *Pain Produced by Chloral Hydrate.* MORGAN. (*New Remedies.*)

M. writes to the *British Med. Journal:* "I have so frequently observed a peculiarity following the use of chloral, which I have not yet seen recorded in any medical book or periodical, that I feel sure that it will be interesting for others, to describe it. In several cases where I had given chloral hydrate in ordinary doses (generally where it had been continued for several days at least) a feeling of pain is experienced all over the body, sharper than that of chronic rheumatism, and often so sharp as to make the patient beg for relief. In each case I have found no relief obtained until the chloral was discontinued. It seems to me to be a general hyperæsthesia of the cutaneous nerves, but sometimes localized in one particular spot. Tincture of gelsemium gives relief to the pain sooner than other remedies." J. S. K.

11. *Salicylate of Quinine as an Anti-Pyretic.* BROWN. (*Edinburgh Med. Journal*, Nov., 76.)

In an article on the above subject, accompanied by four temperature charts, Dr. B. says:

This salt is unsoluble in water, but dissolves readily in certain acid solutions, and tolerably easily in cold, still better in hot, alcohol. In the cases in which it has been administered the dose has ranged from 10 to 40 grs., according to the age of the patient and the mode of administration; 30 grs. in most cases being sufficient. Its anti-pyretic action usually begins in about an hour if it has been administered by the mouth, and continues for some hours subsequently. In some cases the temperature has fallen as much as five degrees in the course of three or four hours. Simultaneously with this anti-pyresis the pulse becomes slower and softer, the respirations also diminish in frequency, the skin becomes cool and moist, and generally the condition of the patient is much improved. He expresses himself as cool and comfortable, and sleep usually follows.

The action of salicylate of quinine is free from the profuse sweating which attends that of salicylate of soda; and, on the other hand, the ringing in the ears and deafness which are occasioned by free doses of sulphate of quinine are hardly noticed after the administration of salicylate of quinine if an over dose be not given. The vomiting which large doses of sulphate of quinine so often produce is entirely absent after the exhibition of salicylate of quinine, nor has albuminuria ever been noticed.

With regard to the mode of administration, it may be given by the mouth either dry (in wafer paper) or suspended in mucilage or in water; but in severe cases, especially in enteric fever, it is best given as an enema, suspended in mucilage, with a small quantity of laudanum to soothe the mucous membrane of the rectum. In this case its action is not so rapid as when given by the mouth, but in about two hours the effect becomes apparent. J. S. K.

12. *Salicin in Chronic Diarrhœa.* (*Am. Med. Bi-Weekly*, Jan. 6, 77.)

In a paper read before the Medical Society of the County of Kings, Brooklyn, N. Y., Dr. Mattison speaks most highly of salicin in chronic diarrhœa; quoting numerous successful cases of treatment.

The doctor considers salicin, tonic, astringent, antiseptic, and anti-fermentive, anti-periodic and finally alterative. It can be administered in pill, powder or solution. To adults in doses of five grains each four hours; to children under two years in doses of from one-half to two grains. Where improvement is not noticed within a week increased frequency of dose is advised. Careful attention to dietetic details and hygenic surroundings should always be observed.

The doctor commends salicin as a drug of unequaled power in chronic diarrhœa, and his success and earnest advocacy warrant a fair trial of the medicine by the profession. J. S. K.

